# Calcium, Phosphate, and Vitamin D Status in Patients with Sarcoidosis—Associations with Disease Activity and Symptoms

**DOI:** 10.3390/jcm12144745

**Published:** 2023-07-18

**Authors:** Łukasz Gwadera, Adam J. Białas, Anna Kumor-Kisielewska, Joanna Miłkowska-Dymanowska, Sebastian Majewski, Wojciech J. Piotrowski

**Affiliations:** Department of Pneumology, Medical University of Lodz, 90-153 Lodz, Poland

**Keywords:** sarcoidosis, calcium, vitamin D, phosphate, life quality, fatigue

## Abstract

Sarcoidosis is a systemic granulomatous disease with a variety of presentations. One of the known symptoms are altered vitamin D metabolism and hypercalcemia. In our study, we aimed to assess associations between disease activity, inflammatory parameters, and vitamin D and calcium status. The secondary aim was to find any dependencies between calcium and vitamin D metabolism and fatigue and quality of life in patients with sarcoidosis. We enrolled 58 patients with sarcoidosis (47 classified as active disease, 11 classified as non-active) and compared them with 25 healthy volunteers. Calcium concentration was significantly higher in the study group than in healthy controls. It correlated with some inflammatory markers but not with vitamin D status. Not calcium nor vitamin D, but phosphate concentration correlated with life quality was assessed with the use of the Sarcoidosis Health Questionnaire. An association between phosphate concentration and fatigue was also noted, but it did not reach statistical significance. Calcium concentration was higher in patients with sarcoidosis, but it was not an indicator of the disease activity, while phosphate concentration was significantly lower in patients with active sarcoidosis.

## 1. Introduction

Sarcoidosis is a granulomatous disease that can potentially affect every organ; thus, the presentation and natural history may vary strongly among the patients. Its etiology is yet to be fully explained and only a part of pathophysiological mechanisms is already known. In most of the cases, sarcoidosis is a self-limiting condition; nonetheless, it can significantly affect the quality of life. In sarcoidosis patients, alterations in vitamin D3 metabolism are frequently present. Activated macrophages, due to the increased activity of 1-alpha hydroxylase, transform 25(OH)D3 into 1,25(OH)_2_D3 (the more active form) without strict control and regardless of calcium concentration. This finally may lead to hypercalcemia as well as hypercalciuria [1,2,3]. One of the common complaints, even in cases of the disease remission, is chronic fatigue—not life-threatening, but a debilitating syndrome. The potential influence of vitamin D status on chronic fatigue seems probable as vitamin D has its role, for example, in the course of fibromyalgia—a condition sharing many similarities with chronic fatigue [4].

Our study aimed to find associations between the activity of sarcoidosis, evaluated with the use of clinical, biochemical, and lung function parameters, and vitamin D3 and calcium (Ca) metabolism. The secondary objective was to assess if vitamin D3 status would influence fatigue, as well as the quality of life of patients with sarcoidosis.

## 2. Materials and Methods

### 2.1. Participants

Participants were recruited from the inpatient pulmonary ward and outpatient clinic of the Barlicki Memorial University Teaching Hospital. The recruitment process took place between November 2016 and March 2020. A major criterion of inclusion was a diagnosis of sarcoidosis made in the past or during the present hospitalization, according to current guidelines [5,6]. Except for cases of Löfgren syndrome, histopathological confirmation of sarcoidosis was preferred; however, in some cases, clinical picture accompanied by typical findings in the radiological examination after the exclusion of other possible diagnoses were sufficient. The activity of the disease, as well as the treatment, were not discriminated at the recruitment stage. Only sarcoidosis of the heart with clinical signs of heart failure and neurosarcoidosis were excluded due to their potential to influence directly cognitive functions, effort tolerance, and fatigue. Other forms of extrapulmonary sarcoidosis did not disqualify patients. As exclusion criteria, we established comorbidities presenting with fatigue, exercise intolerance, cognitive function impairment (e.g., depression, dementia, heart failure more severe than NYHA II and renal failure), autoimmunological diseases, neoplasm in medical history, recent stressful life situations, vitamin D and Ca supplements intake. Patients were divided into two subgroups depending on the disease’s clinical activity. As clinically active sarcoidosis, we recognized a case meeting at least one of following criteria: Löfgren syndrome present at the time of enrollment or in the last 3 months, symptomatic pulmonary or extrapulmonary sarcoidosis, hypercalcemia or hypercalciuria (without other probable cause), radiological progression, worsening of lung function tests results or increased lymphocyte percentage in bronchoalveolar lavage fluid (BALf). The clinically inactive disease was diagnosed when there was radiological and clinical remission or, in the case of radiological stabilization, in a patient without any signs and symptoms of pulmonary and extrapulmonary sarcoidosis.

The control group was recruited from age- and sex-matched healthy volunteers.

### 2.2. Data Collection

Data concerning demography, comorbidities, presentation of sarcoidosis, radiological stage, basic biochemical blood parameters, and lung function tests (spirometry, TLCO) routinely performed during the diagnostic process or follow-up visit were collected in the study group. Additional blood samples had been taken and after preparation—they had been stored at −80 Celsius. They were then used for measurements of interferon-gamma (INF-gamma), soluble interleukin 2 receptors (sIL-2R), neopterin, angiotensin-converting enzyme (ACE), 25(OH)D3, 1,25(OH)_2_D3 with use of the ELISA and high sensitivity CRP (hsCRP) measurement with turbidimetric methods. The kits used to perform measurements are listed in the Supplementary Materials. Additionally, fatigue and life quality were assessed with the use of utility-proofed questionnaires, namely the Fatigue Assessment Scale (FAS) (https://www.wasog.org/dynamic/media/78/documents/Questionairres/679743_FAS_Polish.pdf, accessed on 1 December 2016) and Sarcoidosis Health Questionnaire (SHQ) [7,8,9].

In the control group, basic demographical data and medical history were noted and blood samples according to the same protocol as in the study group were taken and stored.

### 2.3. Statistical Analysis

Continuous data were presented as mean with standard deviation (SD) or quartiles and interquartile range (IQR), from LQ (25%) to UQ (75%), depending on the data normality. Variables were compared using the unpaired Student’s *t*-test, Welch *t*-test, or the Wilcoxon rank sum test with continuity correction, depending on data normality and homogeneity of variance. Differences among more than two groups were analyzed using the Kruskal–Wallis rank sum test or one-way ANOVA, with correction for multiple comparisons performed using the Bonferroni method. The correlation analysis was performed using Spearman’s rank correlation rho.

Categorical data were presented as absolute values and percentages. Such data were compared using Pearson’s Chi-square test (with Yates correction if appropriate) or Fisher’s exact test concordantly with test assumptions.

A comparison in biochemical parameters and FAS between the control and the study group and between the active and the non-active sarcoidosis group was made. In the subgroups of sarcoidosis patients (active vs. non-active), we also compared lung function parameters and quality of life.

For a more comprehensive analysis, a logistic regression model was generated. The sarcoidosis activity was used as an outcome and the clinical features (demographics, clinical characteristics of the disease, pulmonary function test results, blood count parameters, immunological markers, biochemical tests results, BALf, FAS, SHQ results and treatment) as predictors. First, the multivariate model that included all selected predictors was estimated, and then forward and backward stepwise selection approaches were used to restrict the model. The lowest Akaike information criterion (AIC) value was chosen as the determinant of the best final model. Receiver operating characteristics (ROC) and area under the curve (AUC) analysis were performed to determine the predictive power of the model.

Analysis was performed using the R software ver. 4.3.1 (R Core Team 2018). R: A language and environment for statistical computing. R Foundation for Statistical Computing, Vienna, Austria).

## 3. Results

### 3.1. Participant’s Characteristic

Of 103 patients enrolled, 58 were finally qualified for further analysis. Additional exclusions were made as patients treated with steroids and other immunosuppressive medications were finally disqualified. Furthermore, incomplete material banking resulted in a decrease in the final number of the study group participants. The control group consisted of 25 healthy volunteers. There were no significant between-group differences in median age (*p* = 0.06), sex (*p* = 0.08), or ethnicity—all participants were Caucasians. Details are shown in Table 1.

### 3.2. Control vs. Study Group

When comparing the control with the study group, statistically significant differences could be found in the concentration of the ACE median, 799.85 vs. 1588.02 ng/mL (*p* = 0.0005); sIL-2R, 3.01 vs. 8.70 pg/mL (*p* = 0.0002); INF-gamma, 9.40 vs. 11.93 pg/mL (*p* = 0.005); and hsCRP, 0.34 vs. 2.76 mg/L (*p* = 0.01). The relevant difference in Ca concentration was also noted with higher values in the study group: median, 2.14 vs. 2.42 mmol/L (*p* < 0.0001). The level of both forms of vitamin D3—25(OH)D3 and 1,25(OH)_2_D3—did not vary between healthy controls and sarcoidosis patients (*p* = 0.36 and 0.53, respectively). We also compared a 1,25(OH)_2_D3 to 25(OH)D3 ratio, but no between-group difference was noted. Similarly, parathormone (PTH) and phosphate (P) concentrations were not significantly different. The fatigue score measured with FAS was higher in the study than in the control group (only 22 FAS questionnaires from the study group were available). Details are shown in Table 2.

### 3.3. Active vs. Non-Active Group

A comparison between patients with clinically active and non-active sarcoidosis was also done. Age was not significantly different but mean body mass was higher in the active group, 95.5 vs. 85.5 kg (*p* = 0.01106). The groups did not differ according to reported comorbidities: congestive heart failure (*p* = 1.0), arterial hypertension (*p* = 0.052), nephrolithiasis (*p* = 0.32), and diabetes mellitus (*p* = 0.35), whereas we observed differences according to history of extrapulmonary manifestations (*p* = 0.04), including cardiac involvement (*p* = 0.047). Occurrence of Löfgren syndrome was comparable in patients with active and non-active sarcoidosis (*p* = 0.73). Both subgroups did not vary in lung function parameters (FEV_1_, FVC, TLCO, RV and TLC; RV and TLC were assessed during TLCO measurement). Significantly higher concentrations of CRP (but not hsCRP), 3.0 vs. 6.3 mg/L (*p* = 0.03327); INF-gamma, 9.94 vs. 12.74 pg/mL (*p* = 0.01606); neopterin, 5.20 vs. 8.82 nmol/L (*p* = 0.02139); and sIL-2R, 3.52 vs. 9.44 pg/mL (*p* = 0.0002342) were observed in patients with active sarcoidosis. The ACE concentration did not depend on the activity of sarcoidosis. Concentrations of Ca, both forms of vitamin D3, as well as its ratio were comparable in both groups. The PTH concentration tended to be higher in the non-active group, but it did not reach statistical significance, 34.18 vs. 27.35 pg/mL (*p* = 0.07414). What was interesting was that it was not Ca, but P which varied significantly in patients with active and non-active sarcoidosis—0.75 vs. 0.88 (*p* = 0.03). In addition, statistically significant findings were the differences in BALf lymphocytes percentage, 10.62 vs. 41.1% (*p* = 0.0004378); peripheral blood monocyte count, 0.55 vs. 0.7 103/μL (*p* < 0.007037); red blood cell count, 4.62 vs. 5.10 × 10^6^/μL (*p* = 0.002434); and creatinine level, 0.78 vs. 0.89 mg/dL (*p* = 0.03551), respectively, in the non-active and the active group. No relevant differences could be found in the severity of fatigue and SHQ score. However, only 22 (7 in the non-active and 15 in the active group) questionnaires were available for analysis. The results are summed up in Table 3.

The Ca concentration did not correlate with the vitamin D3 level. Weak correlations were noted between the Ca concentration and ACE, r = 0.39 (*p* = 001); sIL2-R, r = 0.36 (*p* = 0.003); hsCRP, r = 0.32 (*p* = 0.009), but not with the INF-gamma (*p* = 0.096) and neopterin (*p* = 0.23) concentrations. No associations could be found between the levels of Ca, vitamin D3, and quality of life and fatigue. A very interesting finding was the correlation between the P concentration and emotional—r = 0.554 (*p* = 0.00604)—and daily functioning—r = 0.469 (*p* = 0.0239)—aspects of life quality measured with SHQ. Some associations between the P concentration and FAS and other domains of SHQ were noted, but they did not reach statistical significance. The P concentration correlated also with the BALf lymphocyte percentage r = −0.45 (*p* = 0.008), and neopterin r = −0.33 (*p* = 0.006). Data shown in Table 4.

### 3.4. Control vs. Non-Active Group

When comparing the non-active and the control group, no significant differences could be found in the concentration of inflammatory biomarkers. What is interesting, the Ca concentration was significantly higher in the non-active than in the control group—2.41 vs. 2.14 mmol/L (*p* < 0.00001)—despite the lack of significant differences in concentrations of vitamin D, PTH, and P. A higher fatigue score in the non-active group was another relevant finding (32.29 vs. 21.18, *p* = 0.006). Details are presented in Table 5.

### 3.5. Logistic Regression and Sarcoidosis Activity

In the multivariate logistic regression analysis, the four analyzed parameters retained their significance. Namely, the final model (Table 6) consisted of RBC (*p* = 0.02), P (*p* = 0.05), extrapulmonary manifestation (*p* = 0.04), and monocytes (*p* = 0.04).

**Figure 1:**
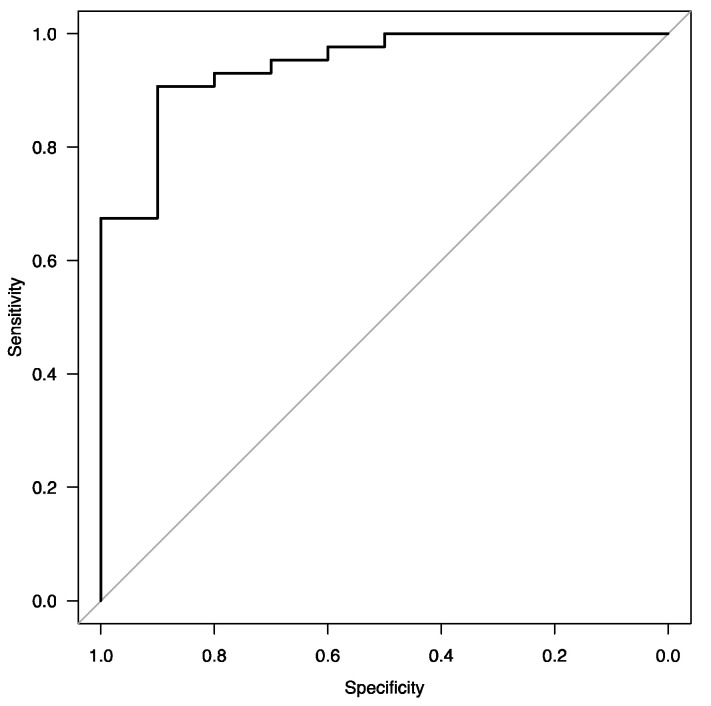
ROC curve of the final model for assessing factors associated with sarcoidosis activity.

A ROC analysis for the model yielded an AUC of 0.94 (95% CI: 0.87–1.0). The ROC curve is shown in Figure 1.

## 4. Discussion

We aimed to find associations between sarcoidosis activity, its clinical presentation, and calcium and vitamin D3 status. The definition of sarcoidosis activity is still a matter of discussion, as there are no simple criteria for assessment. To make the evaluation more precise, we used not only clinical criteria and basic biochemical parameters, but also we measured ACE, sIL-2R, INF-gamma, hsCRP, and neopterin. ACE is one of the first biomarkers used in diagnosing sarcoidosis. Over time, its sensitivity and specificity have been questioned. Some studies find associations between ACE concentration and granuloma burden and radiological presentation of sarcoidosis. However, Popevic et al., in their study, questioned the value of ACE as a marker of activity when compared with the PET-CT assessment [10]. It was also shown that the serum ACE level does not correlate with BALf macrophages activation [11]. Moreover, other granulomatous diseases, genetic predispositions, and drugs can influence ACE concentrations [12]. Our results seem to be consistent with those findings, as there was a significant difference between the control and the study group. Differences between the active and the non-active, as well as the non-active and the control group, were not statistically significant. It seems that ACE may not be an accurate indicator of sarcoidosis activity. Other markers, such as CRP, INF-gamma, neopterin, and especially sIL-2R (with the most significant between-groups difference), seem to reflect disease activity as their concentrations were higher in the active than in the non-active group, and no differences could be found between the non-active and the control group. In the context of sIL-2R, our observation was similar to a study by Lawrence et al., which showed the dependence between sarcoidosis activity, treatment, and sIL-2R concentration [13]. sIL-2R is considered to be useful in diagnosing granulomatous diseases and it is highly specific for sarcoidosis-related uveitis [14,15]. Some data suggest that it can have a prognostic value [16]. It appears, however, that in the case of differential diagnosis in patients with low activity of the disease, usefulness of sIL-2R is questionable. An association between sarcoidosis activity and response to immunosuppressive treatment and INF-gamma inducible chemokines was found by Su et al. [17]. Additionally, Prior and Haslam showed elevated INF-gamma concentration in patients with active sarcoidosis, and its decrease after treatment with corticosteroids [18]. Our data were consistent with those findings, as INF-gamma concentration was elevated in patients with active sarcoidosis, while in non-active sarcoidosis it was comparable with healthy controls. Interestingly CRP, not hsCRP, varied significantly between the non-active and the active sarcoidosis group. As monocytes are key cells in forming granulomas, a higher monocyte count in peripheral blood in patients with active sarcoidosis seems to be consistent with disease pathobiology. A surprising finding was higher RBC in the active than in the non-active sarcoidosis group. The reason for such a phenomenon is unclear. Inflammation, especially chronic, is associated with anemia. Potentially, the activation of macrophages, which plays an essential role also in erythropoiesis [19], may have a certain meaning. Although none of the study group participants had a diagnosis of kidney disease or renal sarcoidosis, the mean creatinine concentration was slightly higher in patients with active sarcoidosis than in those with the non-active disease. This observation may have several explanations. One of the reasons is the higher mean body mass of the active group participants, while body mass correlates with serum creatinine concentration [20,21]. Another cause could be hypercalcemia, which may influence kidney functioning; however, no correlation between Ca and creatine concentration could be found. Also, inflammation itself could be a cause. CRP was proven to be associated with renal filtration impairment [22]. Obviously, an increased percentage of lymphocytes in BALf was observed in the active sarcoidosis group, which was an expected finding, because it is considered to be one of the markers of the disease activity.

Data concerning vitamin D status in sarcoidosis patients and its association with disease activity and prognosis remains incomplete. Some authors found associations between 25(OH)D3 deficiency and an increased activity of the disease and poorer prognosis [23,24]. Others found increased 1,25(OH)_2_D3 in patients with a more severe course of sarcoidosis [25,26]. Additionally, the matter of vitamin D supplementation in sarcoidosis patients is still questionable. Because increased conversion of 25(OH)D3 into its more active form—1,25(OH)_2_D3—is thought to be a pivotal mechanism of hypercalcemia, we measured not only both forms of vitamin D3, but we also compared the 25(OH)D3 to 1,25(OH)_2_D3 ratio. We suspected that this would be a more sensitive indicator of granuloma mass and activity, while vitamin D concentration is dependent on many factors and its normal range in the population is wide. Despite the statistically significant difference in concentration of Ca between the study and the control group, no differences could be found in levels of 25(OH)D3, 1,25(OH)_2_D3, nor its ratio. What is more, Ca concentration did not correlate with any form of vitamin D3 concentration. Our findings supported the opinion that patients with sarcoidosis have higher mean Ca concentrations (Ca even correlated with ACE level), but it is not a good indicator of disease activity except in cases of hypercalcemia. Interestingly, there was no correlation between INF-gamma and calcium. It is an unexpected finding, while INF-gamma is one of the major activators of macrophages, which are considered to be responsible for the overproduction of 1,25(OH)_2_D3 and hypercalcemia. Ca levels correlated with sIL-2R. sIL-2R probably also takes part in macrophage activation and is a marker of immunological response [15]. This is consistent with the study of Lawrence et al., who observed the highest concentration of sIL-2R in patients with hypercalcemia [27]. Vitamin D3 seems not to be a useful marker of disease activity. PTH and P were other parameters we measured, as they are inseparable elements of Ca metabolism. We suspected that change in PTH and P would be a sensitive feedback mechanism to disturbances in Ca concentration. What is more, the normal range for PTH is much better defined than for vitamin D3 in the general population. Interestingly, our data suggest that patients with active sarcoidosis have significantly lower P concentration than those with the inactive disease. As those patients tended to have lower PTH levels and no significant difference in vitamin D status was found, to explain this finding, we needed to refer to the complexity of the P metabolism and the role of FGF23 (tightly connected with 1,25(OH)_2_D3) and other phosphatonis [28]. First of all, inflammation itself may influence the FGF23 level. Another relevant issue may be the potential direct role of the lungs in P overturn. It is known that type IIb of the Na+/Pi co-transporter is expressed in the lungs and its clinical relevance is proved, as its inactivation may lead to pulmonary alveolar microlithiasis [29]. In our study, the P concentration correlated with neopterin and correlated negatively with BALf lymphocyte percentage, which would support the possible role of those mechanisms in alterations in P metabolism.

One of the most interesting findings of our study was that the P concentration was the only parameter, which correlated with scoring in certain domains of life quality measured with SHQ. Additionally, a tendency towards a lower P and more intensive fatigue was noted, but it did not reach statistical significance. The literature on this matter is scarce. The best-known association between P and fatigue is the accumulation of inorganic phosphate in muscles during effort and its role in mediating sensation of muscle fatigue [30]. Interestingly, the study by Harper et al. showed a significantly lower P level in the white matter of patients with major depressive disorder compared to healthy controls, and a significant negative correlation between the P level and the symptoms’ intensity, which is explained by altered bioenergetic metabolites turnover [31]. Kattenbach et al. found altered brain activation in patients with chronic fatigue [32]. Altogether, those findings may support the role of P in fatigue pathophysiology. What is more, fatigue is one of the complaints of patients with hypophosphatemia caused by other congenital and acquired conditions [33]. However, significance of this finding needs further study. Neither sarcoidosis activity nor inflammatory markers concentration were associated with fatigue level or life quality. As fatigue may be reported by some patients months, sometimes even years, after complete radiological remission of the sarcoidosis, the etiology of chronic fatigue seems far more complex than just chronic inflammation and direct cytokine action.

The number of participants was the main limitation of our study, especially in the context of fatigue and quality of life assessment. Furthermore, the single-point measurement of the parameters could be considered a disadvantage. Follow-up with reassessment would provide valuable information as a seasonal change in vitamin D status in Polish climate and latitude has material importance. Positron emission tomography (PET) is considered the most objective tool for the assessment of disease activity [34]. The lack of such an assessment is another limitation of the study. We acknowledge that our findings need confirmation in a more numerous study group.

## 5. Conclusions

Calcium concentration in patients with sarcoidosis correlated with several inflammatory markers and ACE, but not with vitamin D3 status and disease activity assessed with the use of typical clinical parameters. Fatigue did not depend on disease activity. No associations between vitamin D3 status and Ca concentration, fatigue and life quality could be found. The P concentration seemed to depend on sarcoidosis activity and was the only parameter correlating with some aspects of SHQ scoring.

## Figures and Tables

**Table 1:** The basic characteristic of the control and the study group.

Characteristic		Control Group	Study Group	*p*
Number		25	58	
Age, median (IQR)		32 (28–41)	39 (34–43)	0.06
Sex	Male	13	42	0.08
	Female	12	16	
		Study group		
		Active sarcoidosis	Non-active sarcoidosis	
Number		47	11	
Sex	Male	36	6	
	Female	11	5	
BMI		30.17 ± 4.47	26.50 ± 3.95	0.0201
Extrapulmonary sarcoidosis in history	total	4	5	0.04
	cardiac	2	3	0.047
History of Löfgren syndrome		22	6	0.73
Comorbidities	Hypertension	7	2	0.052
	Heart failure	1	0	1.0
	Diabetes	1	1	0.35
	Nephrolithiasis	4	2	0.32

**Table 2:** Comparison of ACE, inflammatory parameters, Ca and vitamin D status and FAS in the control and the study group.

Parameter	Control Group	Study Group	*p*
**ACE [ng/mL]**	**798.85 (142.80–1083.10)**	**1588.02 (840.21–2353.40)**	**0.00055**
**IFN-gamma [pg/mL]**	**9.40 (8.54–12.74)**	**11.93 (9.97–17.58)**	**0.005**
Neopterin [nmol/L]	5.89 (2.57–8.46)	7.55 (3.51–16.96)	0.09
**hsCRP [mg/L]**	**0.34 (0–1.28)**	**2.76 (0–13.36)**	**0.01**
**sIL2-R [pg/mL]**	**3.01 (2.43–4.29)**	**8.70 (4.39–14.09)**	**0.0002**
P [mmol/L]	0.79 ± 0.16	0.77 ± 0.21	0.68
**Ca [mmol/L]**	**2.14 (2.09–2.19)**	**2.42 (2.377–2.49)**	**<0.0001**
25(OH)D3 [ng/mL]	63.39 ± 10.36	60.53 ± 13.28	0.36
1,25(OH)_2_D3 [ng/dL]	10.69 (8.04–13.05)	9.39 (7.94–14.47)	0.53
1,25(OH)_2_D3/25(OH)D3	0.17 (0.13–0.22)	0.17 (0.13–0.25)	0.97
PTH [pg/mL]	27.69 ± 10.50	28.69 ± 11.39	0.7088
**FAS**	**21.18 ± 21.18**	**30.36 30.36**	**0.0003**

Data presented as median (IQR) for Wilcoxon rank sum test or as mean ± SD for the *t*-student test. In bold are statistically significant differences.

**Table 3:** Biochemical parameters, lung function tests, fatigue and quality of life comparison in active and non-active sarcoidosis patients.

Parameter	Non-Active Sarcoidosis	Active Sarcoidosis	*p*
Age [y]	39.50 (35.75–42)	39 (32.5–44.5)	0.7885
**Mass [kg]**	**80.5 ± 13.69**	**95.5 ± 16.82**	**0.01106**
Activity biomarkers and basic biochemical parameters			
ACE [ng/mL]	1202.45 (1011.8–2078.9)	1856.7 (728.15–2353.4)	0.6346
**CRP [mg/L]**	**3.0 (1.5–3.7)**	**6.3 (3.2–13.05)**	**0.03327**
hsCRP [mg/L]	1.04 (0.93–2.64)	4.40 (0–19.50)	0.1722
**IFN-gamma [pg/mL]**	**9.94 (9.29–10.05)**	**12.74 (10.80–19.30)**	**0.01606**
**Neopterin [nmol/L]**	**5.20 (0.72–6.87)**	**8.82 (3.87–21.67)**	**0.02139**
**sIL-2R [pg/mL]**	**3.52 (2.36–4.29)**	**9.44 (6.23–14.65)**	**0.0002342**
**Creatinine [mg/dL]**	**0.78 ± 0.08**	**0.89 ± 0.15**	**0.03551**
**Monocyte [10^3^/μL]**	**0.55 (0.42–0.6)**	**0.7 (0.6–0.8)**	**<0.007037**
WBC [10^3^/μL]	6.25 (5.5–7.9)	7.05 (6.07–8.02)	0.6319
**RBC [10^6^/μL]**	**4.62 ± 0.40**	**5.10 ± 0.44**	**0.002434**
**BALf limf. %**	**10.62 ± 2.82**	**41.10 ± 17.23**	**0.0004378**
Calcium, phosphate, and vitamin D3			
Ca [mmol/L]	2.41 (2.36–2.44)	2.43 (2.38–2.49)	0.3802
**P [mmol/L]**	**0.88 ± 0.10**	**0.75 ± 0.22**	**0.03**
25(OH)D3 [ng/mL]	58.59 ± 9.06	61.07 ± 14.28	0.63
1,25(OH)_2_D3 [ng/dL]	8.20 (7.69–13.46)	9.43 (8.15–14.56)	0.7944
1,25(OH)_2_D3/25(OH)D3	0.14 (0.13–0.25)	0.18 (0.13–0.25)	0.833
PTH [pg/mL]	34.18 ± 11.93	27.35 ± 10.97	0.07414
Lung function tests			
FEV1 [L]	3.37 (3.09–4.40)	3.90 (3.40–4.29)	0.7468
FVCexpiratory [L]	4.63 ± 1.11	4.86 ± 4.86	0.5455
FEV_1_/FVC ex z score	−0.43 (−0.68–−0.17)	−0.35 (−0.99–−0.17)	0.904
RV [L]	1.71 (1.65–2.04)	1.86 (1.61–2.16)	0.7427
TLC [L]	6.26 ± 1.08	6.74 ± 1.08	0.2272
RV/TLC	0.30 ± 0.03	0.29 ± 0.07	0.8994
T_L,CO_ [mmol/min/kPa]	9.46 ± 1.64	10.88 ± 2.39	0.09733
Fatigue and life quality			
#FAS	32.29 ± 6.52	29.47 ± 9.24	0.478
#SHQ emotional	4.14 ± 0.51	4.14 ± 1.13	0.9829
#SHQ physical	4.62 ± 1.00	3.81 ± 1.04	0.08476
#SHQ daily functioning	3.86 ± 0.73	3.68 ± 1.25	0.7107
#SHQ total	3.93 (3.64–4.22)	4.14 (3.6–4.6)	0.7779

Data presented as median (IQR) for Wilcoxon rank sum test or as mean ± SD for the *t*-student test. #—*n* = 7 in the non-active and *n* = 15 in the active sarcoidosis group. In bold are statistically significant results.

**Table 4:** Correlations between Ca, P, vitamin D and inflammation markers, life quality, and fatigue.

Parameter	Rho	*p*
**Ca and ACE**	**0.39**	**0.001**
**Ca and hsCRP**	**0.32**	**0.009**
**Ca and sIL2-R**	**0.36**	**0.003**
Ca and INF-gamma	0.21	0.096
Ca and neopterin	0.15	0.23
Ca and creatinie	0.12	0.398
Ca and FAS	0.16	0.47
Ca and SHQtotal	0.20	0.36
**P and BALf lymph. %**	**−0.45**	**0.008**
P and sIL2-R	−0.24	0.05
**P and neopterin**	**−0.33**	**0.006**
P and FAS	−0.39	0.07
P and SHQtotal	0.406	0.0607
**P and SHQdaily**	**0.469**	**0.0239**
**P and SHQemotional**	**0.554**	**0.00604**
P and SHQphysical	0.377	0.0759
FAS and 25(OH)D3	−0.02	0.97
FAS and 1,25(OH)_2_D3	−0.003	0.99
SHQtotal and 25(OH)D3	0.35	0.19
SHQtotal and 1,25(OH)_2_D3	0.02	0.94

Data presented as rho for the Spearman’s rank correlation. In bold are statistically significant correlations.

**Table 5:** Comparison of non-active sarcoidosis patients and healthy controls.

Parameter	Non-Active Group	Control Group	*p*
ACE and inflammatory parameters			
ACE [ng/mL]	1202.45 (1011.8–2078.9)	798.85 (142.8–1083.1)	0.082
INF-gamma [pg/mL]	9.94 (9.29–10.48)	9.40 (8.54–12.74)	1
Neopterin [nmol/L]	5.20 (0.71–6.87)	5.89 (2.57–8.46)	1
hsCRP [mg/L]	1.04 (0.93–2.64)	0.34 (0–1.28)	0.621
sIL2-R [pg/mL]	3.52 (2.36–4.29)	3.01 (2.43–4.29)	1
Calcium, phosphate, and vitamin D			
**Ca [mmol/L]**	**2.41 (2.36–2.44)**	**2.14 (2.09–2.19)**	**<0.00001**
P [mmol/L]	0.88 ± 0.10	0.79 ± 0.16	0.06526
Ca/P	2.73 (2.56–2.93)	2.53 (2.33–3.19)	1
1,25(OH)_2_D3 [ng/dL]	8.2 (7.69–13.46)	10.69 (8.04–13.05)	0.7928
25(OH)D3 [ng/mL]	58.59 ± 9.06	63.39 ± 10.36	0.572
1,25(OH)_2_D3/25(OH)D3	0.14 (0.13–0.25)	0.17 (0.13–0.22)	0.9743
PTH [pg/mL]	34.18 ± 11.93	27.69 ± 10.50	0.174
Fatigue			
**FAS**	**32.29 ± 6.52**	**21.18 ± 6.78**	**0.003**

Data presented as median (IQR) for Kruskal–Wallis or as mean ± SD for the ANOVA. In bold are statistically significant differences.

**Table 6:** Final multivariate regression model for active sarcoidosis.

Coefficient	Estimate	Standard Error	z-Value	*p*
(Intercept)	−33.8	15.33	−2.21	0.03
RBC	7.13	3.0	2.38	0.02
P	−6.16	3.18	−1.94	0.05
Monocytes	11.2	5.55	2.018	0.04
Extrapulmonary manifestation	−4.05	1.95	−2.082	0.04

## Data Availability

The data used and/or analyzed in this study are available from the corresponding author on reasonable request.

## Supplementary Materials

The following supporting information can be downloaded at: https://www.mdpi.com/article/10.3390/jcm12144745/s1, Table S1. Kits used for the measurement of the inflammatory parameters and vitamin D.
