# Supplementary material for: Calcium, Phosphate, and Vitamin D Status in Patients with Sarcoidosis—Associations with Disease Activity and Symptoms
Source: J Clin Med. 2023 Jul 18;12(14):4745. doi: 10.3390/jcm12144745 (PMC10381487; doi:10.3390/jcm12144745)
Supplement: Supplementary file 1 [file jcm-12-04745-s001.zip › jcm-2435656-supplementary.pdf]

Table S1. Kits used for the measurement of the inflammatory parameters and vitamin D.

Parameter	Kit producent
ACE	Cloud Clone Corp. USA
INF-gamma	BioVendor Czech Republic
Neopterin	IBL International GmBH
1,25$(OH)_2$D3	Immunodiagnostic System (IDS USA)
25(OH)D3	Immunodiagnostic System (IDS USA)
sIL2-R	Diaclone SAS France
hsCRP	biochemical analyzer Olympus AU 640e Beckman Coulter
